# Co-aggregation of MSC/chondrocyte in a dynamic 3D culture elevates the therapeutic effect of secreted extracellular vesicles on osteoarthritis in a rat model

**DOI:** 10.1038/s41598-022-22592-4

**Published:** 2022-11-18

**Authors:** Abazar Esmaeili, Samaneh Hosseini, Amir Kamali, Maryam Hosseinzadeh, Faezeh Shekari, Mohamadreza Baghaban Eslaminejad

**Affiliations:** 1 Department of Stem Cells and Developmental Biology, Cell Science Research Center, Royan Institute for Stem Cell Biology and Technology, ACECR, Tehran, Iran; 2 Faculty of Sciences and Advanced Technologies in Biology, University of Science and Culture, Tehran, Iran; 3 Department of Cell Engineering, Cell Science Research Center, Royan Institute for Stem Cell Biology and Technology, ACECR, Tehran, Iran

**Keywords:** Stem cells, Mesenchymal stem cells

## Abstract

Extracellular vesicles (EVs) have therapeutic effects on osteoarthritis (OA). Some recent strategies could elevate EV's therapeutic properties including cell aggregation, co-culture, and 3D culture. It seems that a combination of these strategies could augment EV production and therapeutic potential. The current study aims to evaluate the quantity of EV yield and the therapeutic effect of EVs harvested from rabbit mesenchymal stem cells (MSCs) aggregates, chondrocyte aggregates, and their co-aggregates in a dynamic 3D culture in a rat osteoarthritis model. MSC and chondrocytes were aggregated and co-aggregated by spinner flasks, and their conditioned medium was collected. EVs were isolated by size exclusion chromatography and characterized in terms of size, morphology and surface markers. The chondrogenic potential of the MSC-ag, Cho-ag and Co-ag EVs on MSC micromass differentiation in chondrogenic media were assessed by qRT-PCR, histological and immunohistochemical analysis. 50 μg of MSC-ag-EVs, Cho-ag-EVs and Co-ag-EVs was injected intra-articularly per knee of OA models established by monoiodoacetate in rats. After 8 weeks follow up, the knee joints were harvested and analyzed by radiographic, histological and immunohistochemical features. MSC/chondrocyte co-aggregation in comparison to MSC or chondrocyte aggregation could increase EV yield during dynamic 3D culture by spinner flasks. Although MSC-ag-, Cho-ag- and Co-ag-derived EVs could induce chondrogenesis similar to transforming growth factor-beta during in vitro study, Co-ag-EV could more effectively prevent OA progression than MSC-ag- and Cho-ag-EVs. Our study demonstrated that EVs harvested from the co-aggregation of MSCs and chondrocytes could be considered as a new therapeutic potential for OA treatment.

## Introduction

Hyaline cartilage repair using various therapeutic approaches such as pharmaceutical drugs, surgical interventions and cell therapy, although hopeful, has not led to the desired outcomes^1^. Recently, extracellular vesicle (EV) therapy for cartilage repair has been considered as a promising cell-free strategy^2^. However, this approach still needs to be improved in terms of producing the quality and quantity of EVs to achieve a more therapeutic effect for the restoration of cartilage defects with natural properties.

Researchers have initially tried to produce EVs with more effective therapeutic properties by selecting the proper cell source^3^. In most studies, the EVs of MSC (MSC-EVs), and in some cases chondrocyte (Cho-EVs) and chondrogenic progenitor cells have been considered for OA treatment^4,5^. Thus far, some research groups have studied the effect of MSC-EVs on OA models with the use of different MSC sources including amniotic fluid stem cells^6^, human embryonic MSCs^7,8^, bone marrow MSCs (BMSCs)^9,10^, infrapatellar fat pad-derived^11^ and other cells. According to studies, BMSCs and articular chondrocytes (ACs) could be the appropriate cells to harvest EVs for OA treatment^2,12–14^. They reported articular Cho-EVs could induce a chondrogenic effect on MSCs^5,15^. BMSCs exhibited more immunomodulatory activity than adipose tissue and Wharton’s jelly MSCs^16^. It has been shown that transplantation of spheroids formed from synovium-derived cells and chondrocytes was also effective in cartilage repair^17^. Cell therapy studies have demonstrated that cellular architectures in the form of aggregation improves a range of biological properties of MSCs, including the ability of differentiation, cell survival and the secretion of therapeutic factors^18^. It seems that the study of the therapeutic effect of EVs secreted by MSCs and ACs aggregates for the treatment of OA could be a valuable strategy.

Many studies have shown improvement in chondrogenic differentiation by co-culture of MSCs and chondrocytes^19,20^. Moreover, other research has proved that chondrocytes induce chondrogenesis of MSCs and, MSCs are effective in reshaping and stimulating the proliferation of chondrocytes^21^. It was suggested that these mutual effects could be through trophic factors and paracrine secretion, especially by EVs^22^. MSC-EVs interceded cartilage repair by increasing chondrocyte proliferation, decreasing apoptosis, and controlling immune reactivity^23^. In contrast, Cho-EVs elevated the proliferation and chondrogenic differentiation of MSCs^15^. Diao et al. suggested that 3D co-culture of human MSCs and ACs is mutually advantageous in their therapeutic potential for OA treatment^24^. It is demonstrated that co-culture of MSCs and ACs could reduce and suppress hypertrophy during chondrogenesis and enhance the functional properties of cartilage^25–27^. According to these studies, it is interesting to investigate the effect of co-aggregation of MSCs/ACs in terms of their EVs’ therapeutic effect for chondrogenic differentiation and treatment of OA.

EVs derived from 3-dimensionally (3D) cultured MSCs provided better therapeutic outcome compared to 2D culture^28^. Recently, Rocha et al. have shown that 3D cellular architecture impacts the microRNA (miR) and protein cargo of EVs^29^. 3D MSC cultures improved small interfering RNA (siRNA) transfer to recipient cells^30^. In addition, dynamic 3D culture could increase EV yield^30,31^. It has been shown that the dynamic MSC 3D culture in an orbital-shake sharply enhanced EV yield^31^. Haraszt et al. have demonstrated that microcarrier-based (3D) MSC cultures in spinner flasks improve EV yield and siRNA transfer^30^. Spinner flask culture in addition to inducing de-differentiation of redifferentiated chondrocytes^32^, could be effective for scale-up of MSC and AC aggregate culture by dynamic collision-based assembly^33^. Despite the proved impact of cells produced by dynamic 3D culture on therapeutic outcomes, there is less evidence on the quality of the EV cargo and the quantitative efficiencies of EVs harvested from the aggregates of MSCs and ACs and their co-aggregates by spinner flasks for OA treatment.

In this study, we selected BMSCs and ACs as the most common EV cell sources with considering the prospect of clinical application potential based on preclinical studies^2,12–14^. We hypothesized that a 3D co-culture strategy in the form of MSC/AC co-aggregation in a spinner flask could highly improve therapeutic properties and yield of EV than the aggregation of MSCs and chondrocytes for OA treatment. Hence, for the first time we harvested EVs from aggregates of rabbit BMSCs and ACs, and their co-aggregates in dynamic 3D culture by spinner flasks. Then, we investigated their chondrogenic effects in the micromass culture as an in vitro model, as well as their therapeutic effect on the OA model in rats.

## Results

### EV characterization

**Figure 1:**
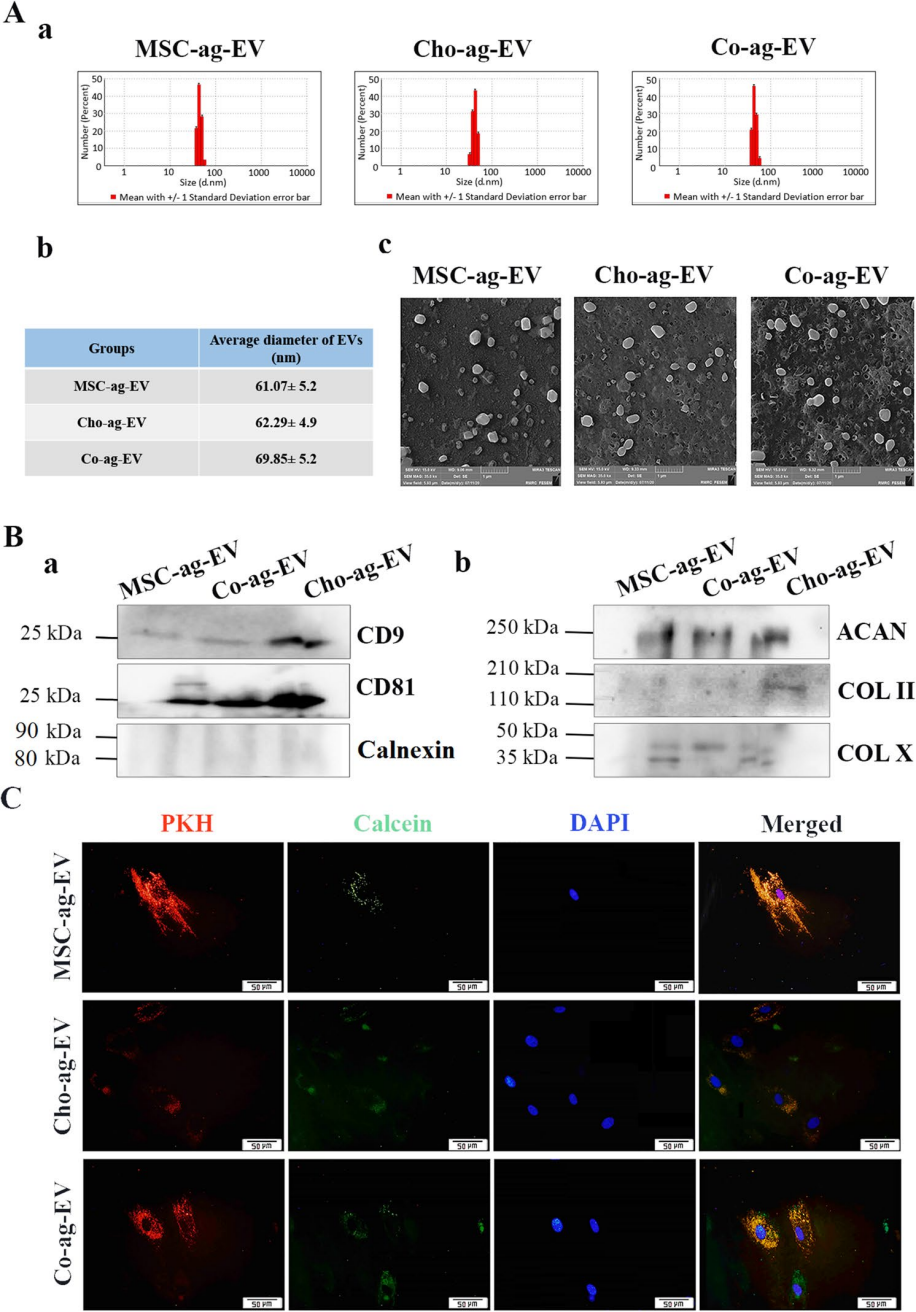
Characterization of extracellular vesicles (EVs) derived from aggregates of, mesenchymal stem cells (MSCs) (MSC-ag-EV), chondrocytes (Cho-ag-EV) and their co-aggregates (Co-ag-EV), and their uptake. (**A**, **a**) Particle size distribution of extracellular vesicles determined by Dynamic Light Scattering (DLS) analysis. (**A**, **b**) Comparison of diameters of harvested EVs. (**A**, **c**) Field Emission Scanning Electron Microscope (FE SEM) of MSC-ag-EV, Cho-ag-EV and Co-ag-EV (Scale bars = 1 μm). (**B**, **a**) Western blot analysis of specific EV surface marker expression including CD9, CD81 and calnexin as a negative control, (**B**, **b**) Western blot analysis of EV proteins, including COL II, ACAN, and COL X. kDa: KiloDalton. (**C**) Uptake of EVs by calcein labeling.

Figure 1 shows characterization of isolated EVs in terms of size, morphology and surface markers. DLS results revealed that the particle size was between 40 and 70 nm in diameter which is compatible with exosome size, and the diameters of the extracted EVs are considerably uniform in all EV groups. The diameter of EVs for the Cho-ag-EVs was 62.29 ± 4. 9 nm, 61.07 ± 5.2 nm for the MSC-ag-EVs and 69.85 ± 5.2 nm for the Co-ag-EV (Fig. 1A, a and b) yet without statistically significant differences. The quantity of harvested EVs from the Co-ag. group (11. 418 μg/Million cell) was more than from the MSCs-ag. (10.058 μg/Million cell) and Cho-ag. (7.984 μg/Million cell) (Table 1). The images of the EVs by the FE-SEM show the vesicular morphology of the EVs for all three EVs groups (Fig. 1A, c). This study shows that the co-aggregation of MSCs/ACs by dynamic 3-D culture in a spinner flask could increase EV yield. EV surface marker proteins including CD9 and CD81 were confirmed by western blots, and calnexin was not observed in isolated EVs (Fig. 1B, a).

**Table 1:** Comparison of the quantity of extracted EVs.

Aggregate or co aggregate	Million cell/ Spinner flask	Protein concentration (μg/ μl)	Extracted volume (μl)	Total protein extracted (μg)	EV/CELL (μg/million cell)
Chondrocyte-ag	175	3.992	350	1397.200	7.984
MSC-ag	130	3.736	350	1307.600	10.05846
Co-ag (MSC + Chondrocyte)	160	5.220	350	1827.000	11.41875

The presence of COL II and AGGRECAN as chondrogenic markers and COL X as a hypertrophic marker in all three groups were proven by Western blotting (Fig. 1B, b). The expression level of COL II and ACAN proteins in all groups were observed to be similar without significant differences (*p* < 0.05). The expression level of the Sox9 protein in all groups had no significant differences, although the Cho-ag-EVs had a higher expression level (*p* < 0.05).

Figure 1C depicts the MSC's internalization of the calcein-labeled EVs after 24 h. Fluorescence microscopy images showed the presence of calcein-labeled EVs in the cytoplasm of the MSCs, which proved that MSCs had internalized the harvested EVs successfully. Uptake was observed in all three groups.

### In vitro evaluation of the chondrogenic potential of isolated EVs

**Figure 2:**
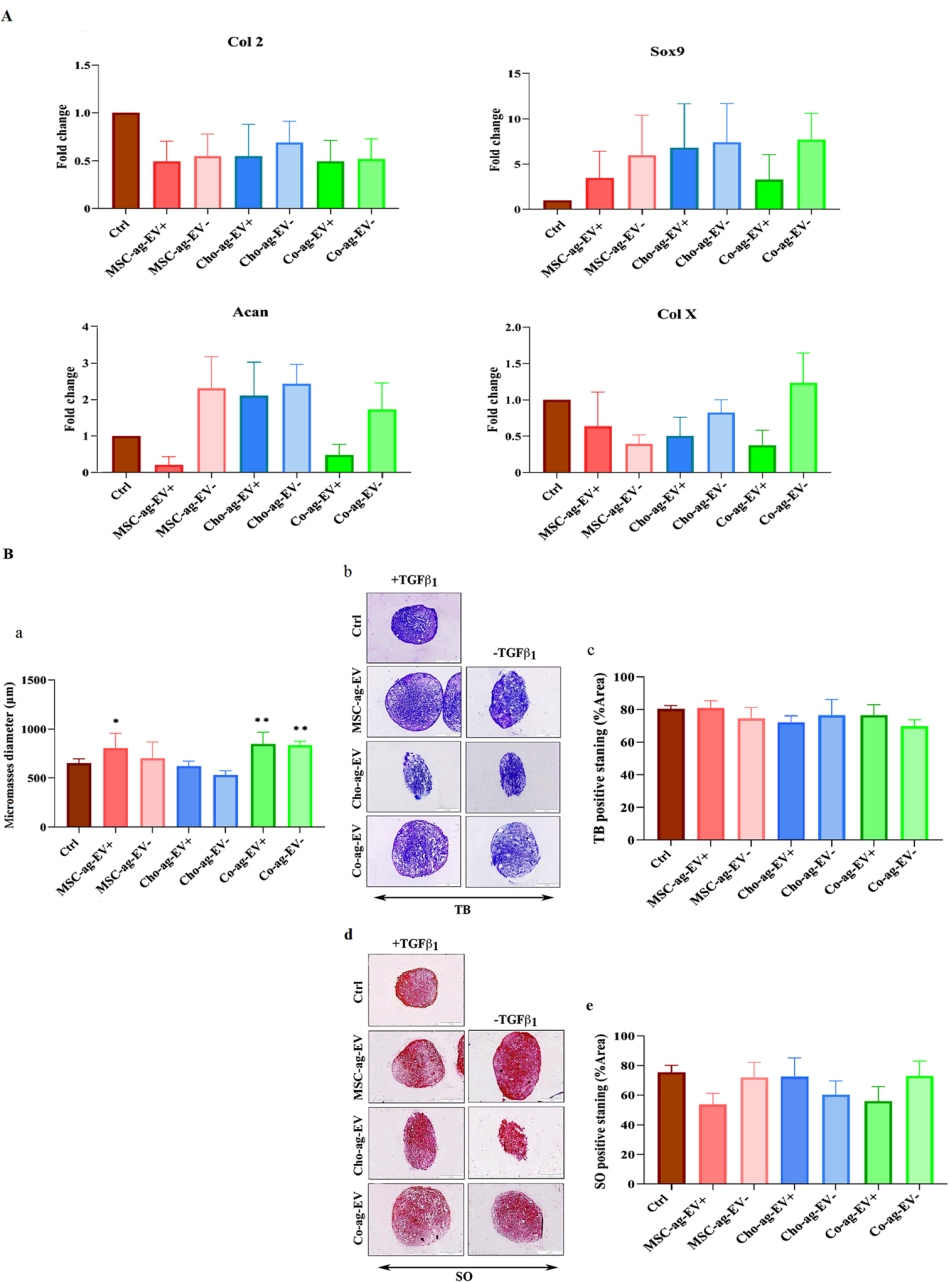
qRT-PCR analysis of chondrogenic genes. (**A**) Histogram displays the qRT-PCR analysis of collagen type II (Col II), Sox9, aggrecan (Acan), and Col X in a cell micromass culture in the existence of extracellular vesicles (EVs) derived from chondrocyte aggregates (Cho-ag-EV) and MSC aggregates (MSC-ag-EV) and Co-aggregates (Co-ag-EV) in chondrogenic medium with ( +) and without (−) TGFß1 after 21 days in vitro histological analysis. (**B**, **a**) Statistical analysis of the comparison between micromass diameters of all experimental groups and the control that demonstrated the Co-ag-EV + and MSC-ag-EV + groups show bigger size and differentiation phenotype of MSCs to chondrocytes than the control group (received chondrogenic medium contain TGFß1) and Cho-ag-EV group. (**B**, **b**) Toluidine blue was performed for (TB) of mesenchymal stem cells (MSCs) micromass sections that differentiated into chondrocytes in the presence of extracellular vesicles (EVs) harvested from chondrocyte aggregates (Cho-ag-EV) and MSC aggregates (MSC-ag-EV) and Co-aggregates (Co-ag-EV) in chondrogenic medium with ( +) and without (−) TGFß1 after 21 days. (**B**, **c**) Statistical analysis of TB staining based on the percent of positive staining area. TB staining does not display a statistically significant difference between the groups. (**B**, **d**) safranin O (SO) staining was performed for all micromass sections. (**B**, **e**) Statistical analysis of SO staining based on the percent of positive staining area. SO staining does not display a statistically significant difference between the groups. The results are expressed as the mean ± SEM. *Significant difference compared with the control group (∗*p* < 0.05; **p < 0.01).

Figure 2 shows the qRT-PCR results for chondrogenic markers in micromass culture after 21 days. We observed downregulation of Col II in all groups treated with EVs (with TGF ß1 ( +) and without TGF ß1(−)), but there was no statistically significant difference among the experimental groups and the positive control group (chondrogenic medium + TGF ß1). The expression level of Sox 9 was enhanced in all groups that received EVs compared to the control group, yet without statistically significant differences. Co-ag-EV− and Cho-ag-EV− had the highest expression level of Sox 9 followed by Cho-ag-EV + and MSC-ag-EV-. ACAN gene expression was upregulated in the Co-ag-EV (+ and −), MSC-ag-EV− and Co-ag-EV−; whereas a decrease in expression level of ACAN was observed in Co-ag-EV + and MSC-ag-EV + compared to the control group. Of noted, no statistically significant differences were found among the groups. We also investigated the expression level of Col X as a hypertrophic marker in chondrogenic differentiation. The Co-ag-EV− group showed an increase in the expression level of Col X, while it was decreased in the other groups in comparison with the control group (Fig. 2A). Histological analysis including measurement of micromass diameters, Safranin O (SO) and Toluidine blue (TB) staining was used to detect the differentiation of BMSCs into chondrocyte lineage. The Co-ag-EV (+ and −) and MSC-ag-EV + groups showed larger micromass size and differentiation of MSCs to chondrocytes phenotypes than the control group (received chondrogenic medium containing TGF ß1) and other groups (Fig. 2B, a). Toluidine blue staining indicated metachromatic characteristics in all groups and the pericellular matrix surrounding the cells inside the lacunae turned purple (Fig. 2B, b). Quantitative results of TB based on the percent of positive staining area did not have a statistically significant difference among the groups (Fig. 2B, c). Similarly, glycosaminoglycan (GAG)-rich spots appeared to be red in color by Safranin O staining (Fig. 2B, d). Quantitative results of SO staining showed no statistically significant differences among the groups (Fig. 2B, e).

**Figure 3:**
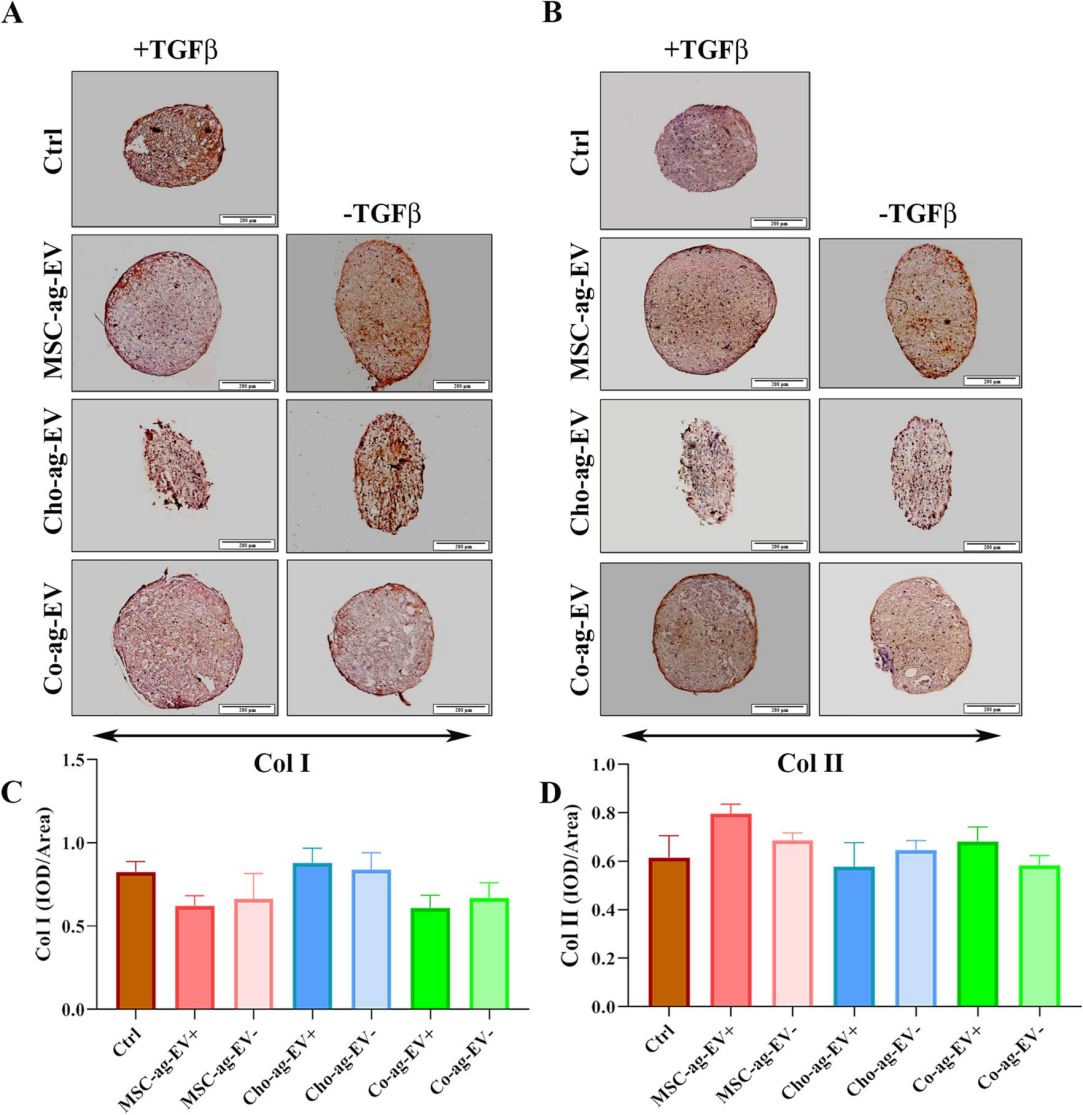
Immunohistochemical analysis of type I collagen (Col I) and type II collagen (Col II). (**A**) IHC staining of Col I and Col II. (**B**) Histograms show the intensity of Col I and Col II expressed in mesenchymal stem cells (MSCs) differentiated into chondrocytes in the presence of extracellular vesicles (EVs) harvested from chondrocyte aggregates (Cho-ag-EV) and MSC aggregates (MSC-ag-EV) and Co-aggregates (Co-ag-EV) in chondrogenic medium with ( +) and without (−) TGFß1 after 21 days. Statistical analysis did not display a statistically significant difference among the groups. The results are expressed as the mean ± SEM. (*p < 0.05).

We also performed immunohistochemical staining for COL I and COL II as a hypertrophic marker and a chondrogenic marker, respectively. Both proteins were detected in all groups and no significant differences were found neither among the groups nor control, except MSC-ag-EV + which showed a higher COL I expression than the control group (Fig. 3).

### Comparison of the effect of EVs on Osteoarthritis treatment

Gait analysis, radiographic examination, histopathology, and immunohistochemistry were performed to evaluate the status of knee healing in rats 12 weeks post EV treatment.

**Figure 4:**
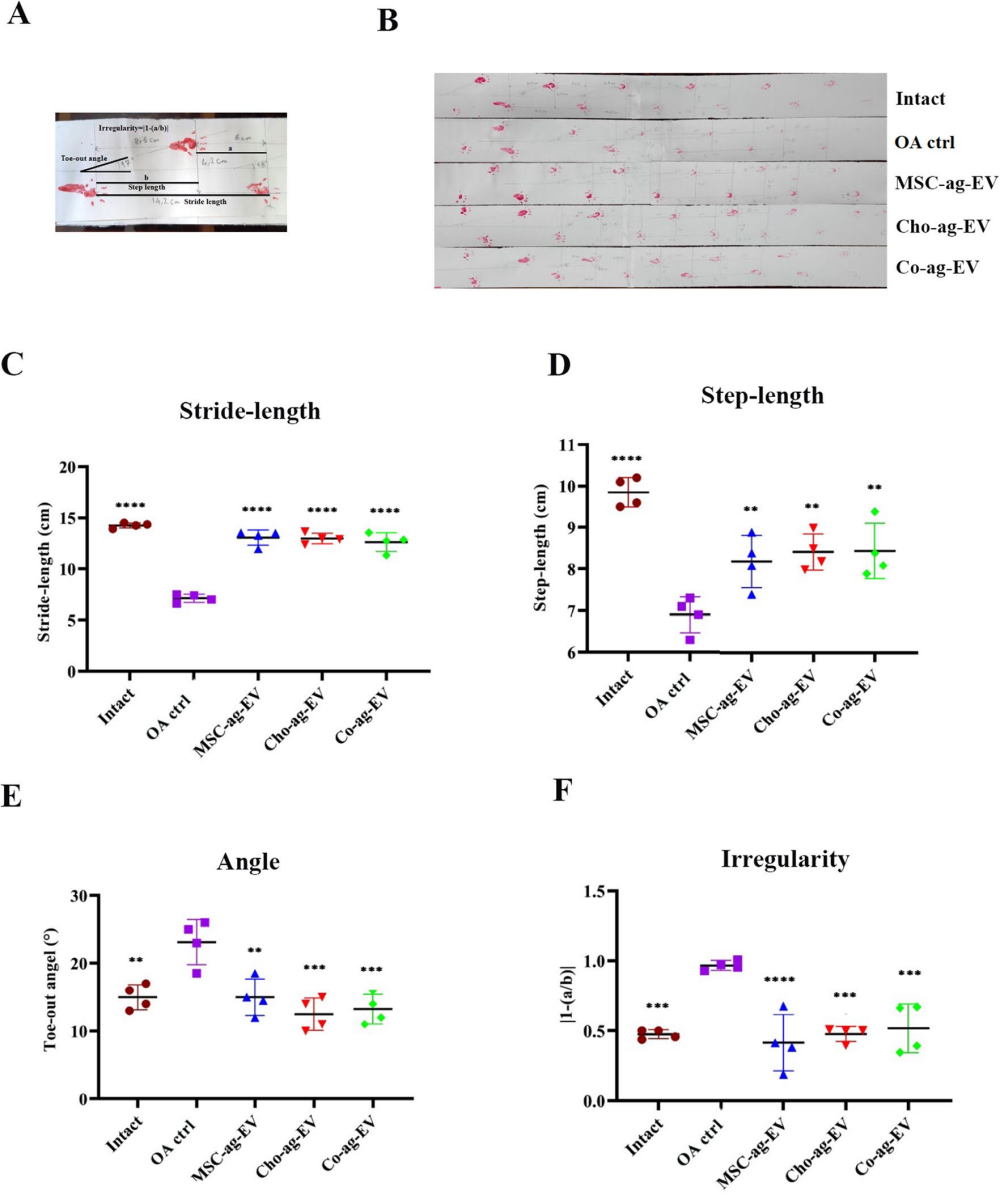
Gait analysis of osteoarthritis (OA) model rats from spatial gate sequences in diverse groups and the effect of the chondrocyte aggregates (Cho-ag-EV) and MSC aggregates (MSC-ag-EV) and Co-aggregates (Co-ag-EV) on recovery of failures in motor function. (**A**) Stride length, step width, toe-out angle, and step irregularity were measured as defined. (**B**) Representation footprints of all five groups. Individual rats with stained hind paws were allowed to walk on the white paper, and their footprints were assessed. (**C**) Shorter stride length (the distance between two foot effects of the same limb), (**D**) step length (the distance between two foot influences of the opposite limb), (**E**) larger toe-out angle, and (**F**) more step irregularity (two unequal consecutive step lengths) represented the severity of joint destruction in that group in comparison with the OA control (OA ctrl) group. The results are expressed as the mean ± SEM. *Significant difference compared with the OA control group; (*p < 0.05; **p < 0.01; ***p < 0.001; and ****p < 0.0001; (n = 5).

Gait analysis was performed to evaluate how the rats walked through the footprint (Fig. 4A-B)^34^. Stride-length significantly enhanced in all EV treated groups in comparison with the OA control group (Fig. 4C) and their results are close to the intact group, especially the MSC-ag-EV group. As expected, Stride-length reduced in the OA group that received no treatment. Similarly, a significant decrease in Step-length was observed in the OA control group compared to the intact, while Step-length enhanced in all EV-treated groups in comparison with the OA control group (Fig. 4D), in a way the increases in Step-length in Co-ag-EV and Cho-ag-EV groups were similar and was more than the MSC-ag-EV group.

A notable increase was observed in the Toe-out angle in OA control rats compared to the intact group. Toe-out angles decreased significantly in all treated groups in comparison with the OA control group, yet similar to the intact group **(**Fig. 4E). Among the EV-treated groups, the Cho-ag-EV group was more close to the intact group (Fig. 4E).

In unilateral injuries in OA model animals, the animal's reluctance to transfer weight from a healthy foot to an injured foot is evaluated through the Irregularity factor (IF). The step irregularity factor improved in all EV-treated groups compared to the OA control group (Fig. 4F).

**Figure 5:**
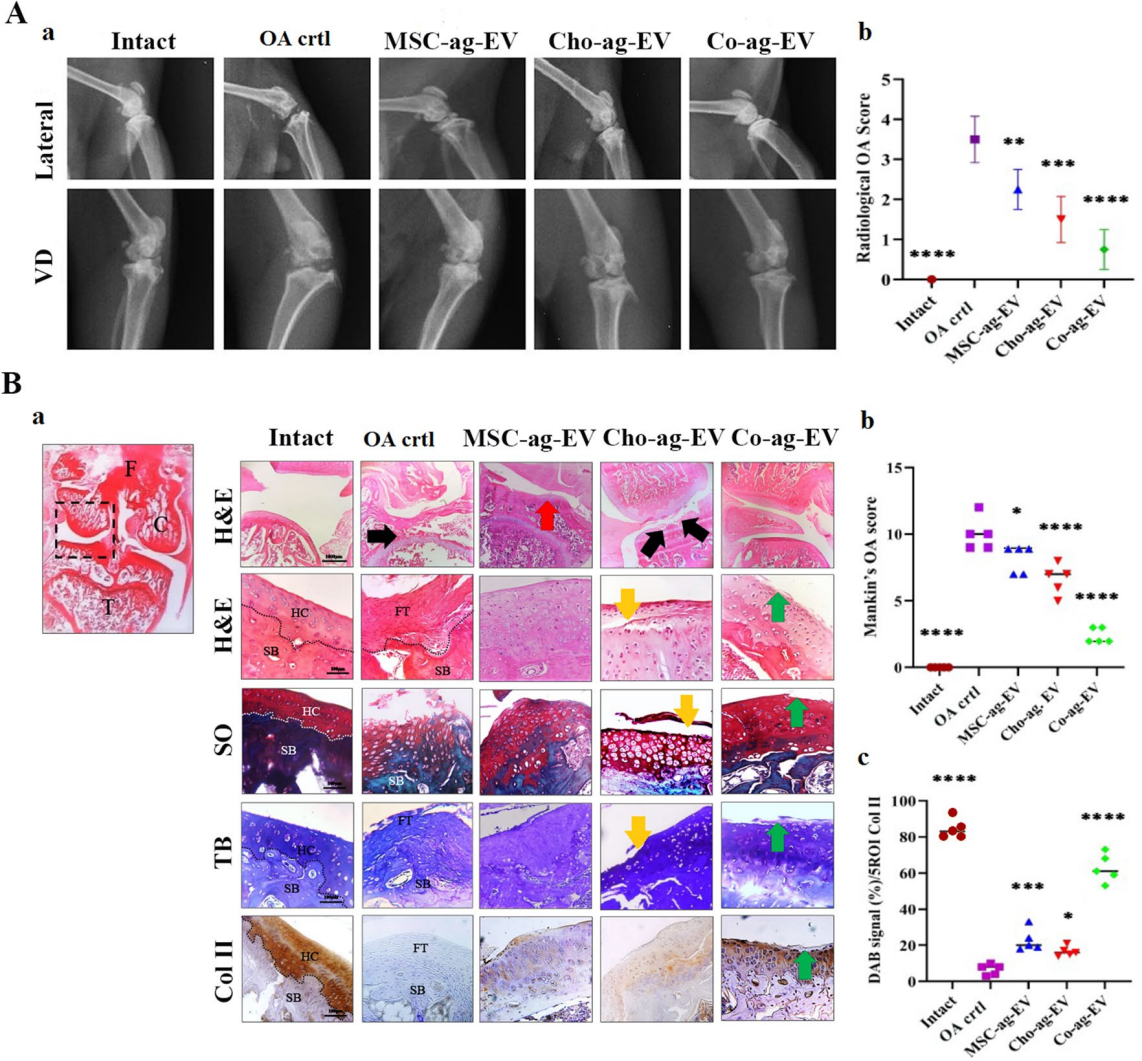
(**A**, **a**) Radiological photograph from lateral and ventrodorsal view (VD) of rats and (**A**, **b**) their statistical analysis. (**B**, **a**) In vitro histological analysis; knee sections were stained using Hematoxylin and Eosin (H&E), Toluidine blue (TB) Safranin O (SO) and immunohistochemical staining for COL II. HC: hyaline cartilage, SB: subchondral bone, FT: fibrous tissue, black arrows: the destruction of articular cartilage, yellow arrow: superficial cleft of articular cartilage, red arrow: thickened articular surface + chondrocytes depletion, green arrows: micro-lesions in articular cartilage surface (scale bars = 100 μm). (**B**, **b**) Mankin’s OA score statistical analysis, (**B**, **c**) DAB signaling (%) 5ROI statistical analysis for COL II. The results are expressed as the mean ± SEM. *Significant difference compared with the OA ctrl (*p < 0.05;  **p < 0.01;  ***p < 0.001; and ****p < 0.0001).

Radiographic assessment (lateral and ventrodorsal view) was accomplished in both experimental and control groups to evaluate the pathologic changes of knee joints after OA induction as well as to observe different EV-treatment effects (Fig. 5A, a). The analysis of the radiological score of various groups showed that the score of all EV-treated groups decreased significantly in comparison with the OA control group (Fig. 5A, b). The radiographs of the intact group exhibited a normal knee joint with the presence of smooth articulating surfaces in the tibial plateau and femoral condyles. In contrast, the radiologic evaluation of the OA control group indicated osteophytes formation, noticeable osteolysis, and deformation of femoral condyles which showed severe OA. Similar findings were also observed in Cho-ag-EV, Co-ag-EV, and MSC-ag-EV treatment groups and different degrees of OA was present in knee joints based on their radiographs. The three treated groups, showed markedly controlled OA development and significant progress of articular damage at the knee joint. Therefore, the radiologic scores of Co-ag-EV treatment was lower than other treatment groups, followed by Cho-ag-EV (Fig. 5A).

Since articular cartilage damage is the main histopathological characteristic of OA joints, the effect of different treatments by Cho-ag-, MSC-ag- and Co-ag-EVs on the histopathological modifications and stringency of articular cartilage damage was assessed using H&E, SO and TB staining in the MIA induced OA model in rats. As depicted in Fig. 5, healthy control (intact) animals displayed normal articular structures with smooth articular cartilage surfaces, normal organization of chondrocytes in hyaline cartilage (HC), intact tide marks and subchondral bone (SB). In contrast, the OA control group presented with massive destruction and loss of articular cartilage, destruction of ligaments and meniscus, osteophyte formation, severe tide mark degradation, articular fibrillation, and permeation of SBs. Although irregularity of articular cartilage surfaces and cartilage degeneration were found in Cho-ag-EV treated animals, this treatment reduced the articular cartilage morphological changes, decreased the permeation of the SBs, and the tide mark degeneration when compared to the OA control group. Histopathological evaluation of MSC-ag-EV treated OA joints revealed a more severe degeneration process in this group in comparison to Cho-ag-EV treated animals with the presence of a thickened chondrocyte depleted articular cartilage with surface irregularities. The SO staining method was used to detect PRGs, as one of the major components of the ECM, in the cartilage. SO staining revealed a marked PRGs depletion in the ECM of the MSC-ag-EV treated OA joints. Administration with Co-ag-EV efficiently reduced the loss of PRGs in the knee joints compared to those of Cho-ag-EV and MSC -ag- EV treated groups. Moreover, the structure of articular cartilage in the Co-ag-EV group showed a close resemblance to intact joints, however, a minimal degeneration of articular cartilage was still evident. SO staining of treated joints in this group displayed the existence of intense PRGs in the ECM (Fig. 5B, a).

The severity of OA damage was numbered using the Mankin’s scoring system (Table 2), and the overall Mankin’s scores were significantly lower in EV-treated groups compared with the OA control group (Table 3). The results showed that OA scores in the Co-ag-EV treatment group were lower than other treatment groups, followed by Cho-ag-EV (Fig. 5B, b).).

**Table 2:** Cartilage value according to the Mankin’s scores system.

Criteria	Score	Histological finding
Structure	0	Smooth intact surface
	1	Slight surface regularity
	2	Pannus/surface fibrillation
	3	Clefts into transitional zone
	4	Clefts into radial zone
	5	Clefts into calcified zone
	6	Total disorganization
Cells	0	Uniform cell distribution
	1	Diffuse cell proliferation
	2	Cell clustering
	3	Cell loss
Toluidin staining	0	Uniform staining
	1	Minor discoloration
	2	Moderate discoloration
	3	Sever discoloration
	4	Total discoloration
Tidemark integrity	0	Intact
	1	Vascularity

**Table 3:** The joint lesions were graded on a scale of 0–14 using the Mankin’s scoring system.

Joints	Intact	OA crtl	MSC-ag-EV	Cho-ag-EV	Co-ag-EV
1	0	10	9	6	2
2	0	9	7	7	3
3	0	12	9	5	2
4	0	10	9	7	2
5	0	9	7	8	3

The comparison of COL II expression proved that the Co-ag-EV treated group can significantly improve COL II expression better than other groups, followed by MSC-ag-EV (Table 4) (Fig. 5B, c).

**Table 4:** DAB signal (%)/5ROI.

ROI	Intact	OA crtl	MSC-ag-EV	Cho-ag-EV	Co-ag-EV
1	83	8	33	16	61
2	80	8	18	21	73
3	93	4	24	14	53
4	85	3	20	17	68
5	80	10	19	15	59

## Discussion

Due to the lack of a self-repairing process for joint hyaline cartilage, a new approach using EVs has recently attracted attention for treatment of cartilage injuries. Improving the therapeutic properties of EVs could affect repairing cartilage injuries. In this study, we attempted to augment the therapeutic properties of EVs by co-aggregation of MSC/AC (3:1). Here, we sought to find a more effective EV source that could better prevent OA progression.

Isolated EVs were characterized by SEM analysis that confirmed the vesicular shape of the isolated EVs in three groups, and DLS showed their size was within a range of 60 to 70 nm which is in accordance with the size of small EVs (exosomes) which have been previously reported^5^. The average diameter of different EV groups did not show a significant difference, which is consistent with a previous report^35^. It should be noted that our group has previously reported the difference between the size of the EVs isolated from chondrocytes and MSCs and that this discrepancy could be related to the difference in the harvesting and isolation methods of the EVs^5^. Our three isolated EV groups expressed the protein tetraspanins surface markers, CD81, and CD9, and they did not express calnexin^36,37^.

Our study showed the co-aggregation of MSCs/ACs during their dynamic 3D culture in a spinner flask increased EVs yield. Accordingly, Cha et al. demonstrated that dynamic 3D culture on the multiwell plate could improve EV yield in comparison to the conventional culture method^31^. It seems co-aggregates (3D co-culture) provide 3D interaction in a 3D culture system between chondrocytes and MSCs and could increase EV production. Moreover, co-aggregation and dynamic 3D culture in spinner flasks are more similar to the natural cartilage niche. Chen et al. have shown that hypoxia-inducible factor 1 alpha (HIF1α) is up-regulated in co-culture pellets in an orbital shaker^38^. HIF1α activation enhances small EV release in human embryonic kidney cells^39^. Altogether, these data demonstrate that in addition to dynamic 3D culture, the HIF1α produced during co-aggregation of MSCs/ACs could probably be effective in increasing EV yield which of course needs further verification.

The results of qRT-PCR showed that all our EV groups have been able to increase the expression of most chondrogenic markers including Col II, Sox9, ACAN and decrease the expression of Col X in the micromasses, though none of these changes were statistically significant in comparison to each other and the positive control group. In general, these data revealed that our different EV- (without TGF ß1) groups could play a similar role to that of TGF ß1 by controlling the expression of genes involved in chondrogenesis and cartilage repair. Therefore, it seems that these EVs could have the potential of stimulating cartilage ECM synthesis and reducing Col X expression and they may be more effective in OA treatment. Accordingly, a previous study reported that isolated EVs from MSCs and chondrocytes 2D monoculture were able to increase the expression of chondrogenic genes in MSC micromasses^5^. EVs have an important effect on the relationship between MSCs and chondrocytes co-culture in order to improve chondrogenesis in the MSCs^22^ such that EVs derived from chondrocytes could promote MSCs differentiation by increasing chondrogenic gene expression and decreasing Col X expression, and MSC-derived EVs could enhance cartilaginous ECM, and matrix glycosaminoglycan synthesis^15,40,41^. In addition, no synergistic effect on chondrogenesis was observed between EVs groups and TGF ß1 during their simultaneous administration. The results of histological analysis of micromasses show that Co-ag-EVs (+ TGF ß1 and − TGF ß1) and MSC + (+ TGF ß1) groups have significantly bigger sizes and a better chondrocyte differentiation phenotype in comparison to the positive control group and other groups. These indicate that these groups have enhanced cell proliferation. Consistently, Yu et al. demonstrated that the MSC EVs stimulate proliferation and chondrogenic differentiation of tendon stem/progenitor cells into mature chondrocytes^42^.

Histological and immunohistochemical analysis of micromass confirmed the RT-PCR results as all isolated EVs induced ECM components synthesis including GAGs by enhancing chondrogenic gene expression, similar to the positive control group. A previous study has proven the existence of TGFβ in AFSC exosome protein content and shown that exosomes counteracted cartilage damage by a positive correlation with their TGFβ content^6^. Another study by Kubosch et al. has shown that the co-culture of chondrocytes with human synovial MSCs (SMSCs) leads to self-organization, chondrogenic differentiation of SMSC, and TGFβ secretion^43^. In addition, researchers demonstrated that TGFβ could stimulate chondrogenesis by provoking the expression of Sox9 and Col II, and TGFβ signaling could lead to further stimulation of chondrocyte proliferation and inhibition of chondrocyte hypertrophy^44,45^. These data justify our results about the increasing diameter of micromass by Co-ag-EV and MSC-ag-EV and their chondrogenic potential on MSC micromass culture, but more research is still required, especially about the existence of TGFβ in Cho-ag-EV. Overall, our in vitro study results showed that all our EV groups have chondrogenic induction potential similar to the positive control group.

We sought to evaluate the therapeutic effect of harvested EVs in vivo on a rat MIA-induced OA model. Gait analysis showed an increase in stride length and step length, and a decrease in irregularity and toe-out angle in all treatment groups in comparison to the OA ctrl group. These data demonstrated that all EV-treated groups could, significantly prevent OA progression. We used radiological images and histopathological studies to precisely address the treatment trend. Although, these findings proved that all EV treated groups could prevent OA progression and help to repair, the Co-ag-EV treated group displayed more therapeutic effectiveness. Administration of Co-ag-EV effectively decreased degeneration of articular cartilage, and promoted ECM synthesis, as consistent with immunohistochemical analysis results. Moreover, Co-ag-EV decreased the inflammatory response of the OA model compared to other groups more effectively. It seems that the use of a higher dose of Co-ag-EVs (than 50 μg /knee joint in rat) will lead to more effective treatment of OA.

Previous studies have shown that MSC-derived EVs induced the migration and proliferation of appropriate types of repairing cells and stimulating cartilage ECM synthesis led to good cartilage repair in the OA model^6–8^. It has been proven that chondrocyte-derived EVs also advance the proliferation and migration of human umbilical cord mesenchymal stem cells^15^, and assist in the chondrogenesis of cartilage progenitor cells^35^, and BMSC^46^. In addition, it is indicated that co-culture could induce MSC chondrogenesis potential with increasing Sox9 gene and protein expression, gene expression of ACAN and COL2A1, and improve GAG production^24^. Moreover, studies have demonstrated that aggregation of MSC improves the anti-inflammatory and immunomodulatory effects potential^47,48^. These data are consistent with our in vitro and in vivo results.

Several mechanisms of action for EVs on OA treatment have been proposed including increasing cell number, bioenergetics effects, and immunomodulation^49^. EVs could also polarize the macrophage phenotype toward M2^50^, and induce the permeation of M2 macrophages into the synovial fluid. They could induce progenitor/stem cell migration, chondrocyte differentiation and proliferation^42^, and inhibit cell apoptosis^23,51^. EVs could also enhance the intracellular ATP level in chondrocytes^50^ which might be related to mitochondria dysfunction in OA^52^. Recent evidence indicates that MSC-EVs could contain mitochondria that could transfer them to chondrocytes^53^. Although the current results clearly showed repair of the OA through controlling the joint inflammatory response and tissue regeneration by our EVs, these regeneration potentials could be attributed to all mentioned mechanisms, but proving them requires more studies.

Moreover, it is demonstrated that 3D co-culture and aggregation could lead to more enrichment of the EVs’ cargo and could elevate their therapeutic effects^24,29,47^. Likewise, it has been proven that all mechanisms of the therapeutic effects of EVs could depend on their cargos^54^, including microRNAs (miRs)^55,56^. According to recent findings, miRs could participate in the control of inflammation, induce GAG expression, suppress chondrocyte hypertrophy, inhibit cell apoptosis, and affect autophagy^57–59^, it seems that the comparison of EV cargo could clear differences between MSC-ag-EV, Cho-ag-EV, and Co-ag-EV miRs composition, and this could the pave the way for more effective EV treatment.

Our in vitro experiments demonstrated that all three groups of isolated EVs have the potential for chondrogenesis induction, and in vivo results proved that Co-ag-EVs could be more effective than others for OA treatment because these EVs contain both benefits of secreted EVs from chondrocytes and MSCs and could be the result of their mutual 3D interaction during 3D culture. Hence, it seems that Co-ag-EVs, in addition, to having higher EV yield, could have more enriched cargo to induce chondrogenesis and have other possible therapeutic effects such as immunomodulation.

## Conclusions

In this study, it was observed that dynamic 3D co-aggregation by spinner flasks, could increase EV yield and elevate the therapeutic effects of harvested EVs. According to our results and considering recent findings, Co-ag-EV cargo probably including TGF ß1 and miRs has more potential for study in order to treat OA by EV. In summary, EV harvesting from co-aggregation by 3D culture, could elevate EV therapeutic properties and provide a new potential for the treatment of diseases including OA.

## Methods

### Aggregates and co-aggregates formation

**Figure 6:**
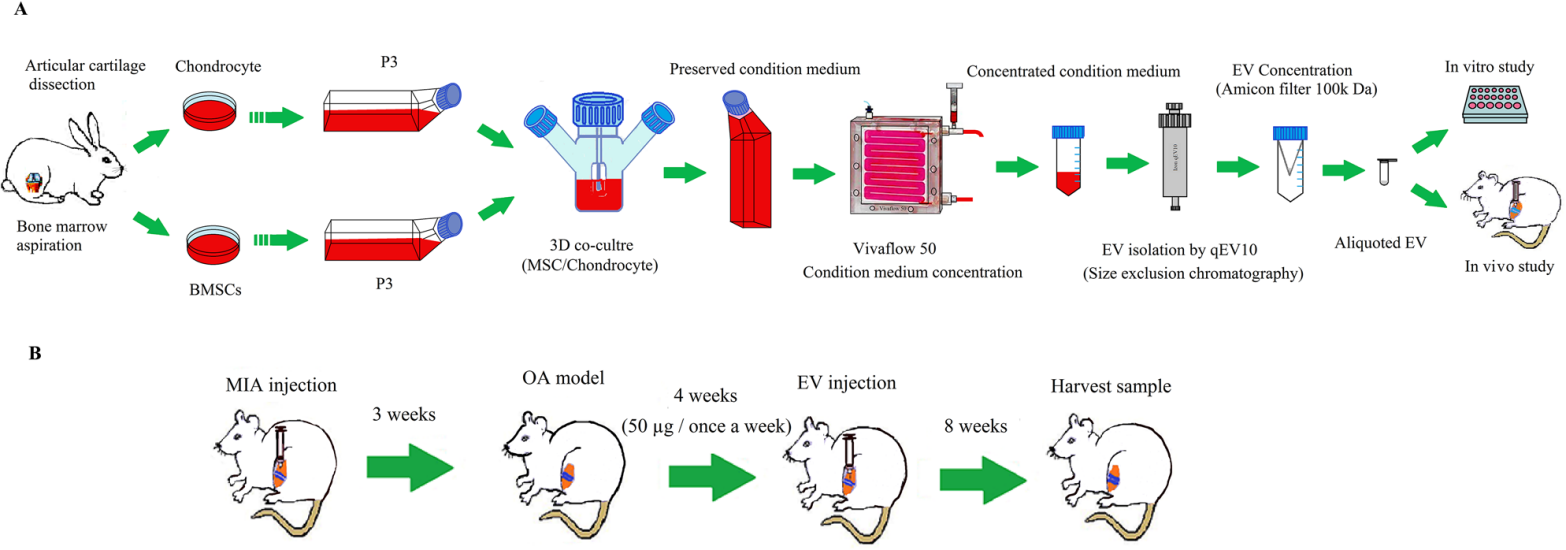
(**A**) Schematic representation of the study process for EV harvesting, and (**B**) in vitro study process.

To induce aggregation, the cells including MSC aggregates, chondrocyte aggregates, and MSC/AC (with ratio 3:1) co-aggregates were suspended at a concentration of 500,000 cells per ml of DMEM medium containing 10% EV- free FBS and transferred to a spinner flask (Integra Biosciences, UK) according to a previous study^33^. Briefly, the spinners were set at 40 rpm for the chondrocytes aggregation and 45 rpm for MSCs aggregation and MSC-AC co-aggregates for 24 h. After that, spinners were set at 60 rpm for 7 days. At day 7, the media was centrifuged (3000 g for 10 min) in order to eliminate cell debris and were then stored in − 80 °C until extracellular vesicles extraction (Fig. 6A).

### Extracellular vesicles isolation

The culture medium was collected from 3D cultures of aggregates in a spinner flask from all experimental groups with a volume of approximately 400 ml. To reduce the batch effect, the collected media was pooled and concentrated by Vivaflow (50 R, 30,000 MWCO Hydrosart-1pcs-Sartorius) to a desired volume (about 30 ml) for size-exclusion chromatography. Fractions containing EVs in PBS were separated by using a qEV10 (70 nm-Izon) column and then re-concentrated with an Amicon Ultra 15 mL centrifugal filter (MWCO 100 k Da) to approximately 350 μl. EV concentration in all three groups was determined by BCA protein assay kit (Millipore, Germany), in accordance with the manufacturer’s instructions, then concentrated EVs were aliquoted and stored at − 80 °C.

### Characterization of extracellular vesicles

EV size distribution and the average diameter of the extracted EVs in all three groups were calculated by dynamic light scattering (DLS) measurement with a Zetasizer (Malvern Instruments, Malvern, UK). The samples from all groups were diluted 1:1000 in PBS to a total volume of 1 ml, and three-time measurement runs were performed with a refractive index of 1.331, the viscosity of 0.9, and a temperature of 25 °C. The resulting data were analyzed by Malvern Zetasizer Software (Malvern Instrument, UK).

For morphological evaluation of EVs, 20 μl of the suspension of fresh EVs (6 μg EV/1 ml PBS) from each group were loaded onto a lane to dry and then coated with gold. The images were acquired with Field Emission Scanning Electron Microscopes (FE SEM, MIRA3, Tescan, Czech).

To check the uptake of EVs by MSCs, MSCs and EVs were labeled by fluorescent dies as previously described^5^. Briefly, we used calcein green fluorescent dye (calcein, AM, c3099, Invitrogen, USA) to assess EVs uptake by MSCs. We added 1 × 10^–6^ M calcein to 10 μg/ml of EVs incubated for 30 min at 37 °C. Then the EVs were resuspended in PBS and centrifuged (at 100 000 × g for 1 h). The MSCs were seeded and incubated at 37 °C to obtain up to 60% confluency. Next, MSCs were labeled with PKH26 (PKH26-Red, PKH26GL, Sigma, Germany) overnight, followed by incubation with calcein-stained EVs. We used DAPI (aqueous DAPI (Fluoroshield) ab104139, Abcam, UK) for staining the nuclei of cells. The stained cells and EVs were fixed and used for imaging (Olympus, IX71; Olympus, Tokyo, Japan).

### Western blot

We suspended 15 μg of EV protein from each sample in sample loading buffer solution at a similar volume and after sonication, heated it to 95 °C for 5 min. All samples were isolated on 12% polyacrylamide gels and then transferred onto an electrophoretic (Bio-Rad) polyvinyl difluoride (PVDF) membrane (Millipore, Germany) (semidry method, 120 V, 75 min). The membrane blocking was performed with 5% bovine serum albumin (BSA) for one hour at room temperature, then incubated overnight at 4 °C by adding specific primary antibodies to CD81 (1: 1000, EX203, Cell Guidance Systems, UK) and CD9 (1: 1000, GTX76182, GeneTex, USA), and calnexin (1: 500, sc-11397, Santa Cruz Biotechnology, USA) as a negative marker for EVs. Then, we added goat anti-mouse HRP-conjugated secondary antibodies (1: 5000, ab6789, Abcam, US) to the membranes and incubated them for two hours at room temperature. Following three-time washing with 1 × TBST (Tris-buffered saline, 0.1% Tween 20), Enhanced Chemiluminescence ECL was added to the blots, and finally the protein bands were observed in a chemiluminescence device (Uvitec, Cambridge, UK). To assess the chondrogenic proteins expression level in the extracted EVs, we also executed a wet western blot analysis with the primary antibodies of COL II (COL2A1, 250,484, Abbiotec, USA), aggrecan (ACAN, NB600-504, Novus Biologicals, USA), and COL X (COL10A, ab58632, Abcam, UK).

### In vitro chondrogenic potential of isolated EVs

A micro-mass culture system in 96 well culture plates was used to enforce MSCs’ chondrogenic differentiation. Briefly, 3 × 10^5^ cells at passage number3 were cultured in chondrogenic medium comprising high glucose DMEM supplemented by 10 ng/ml transforming growth factor-beta 1 (TGF ß1), 1:100 diluted insulin transferrin selenium + premix (Sigma, Germany), 6.25 μg/ml insulin, 6.25 ng/ml selenious acid, 6.25 μg/ml transferrin, 1:100 NEAA, and 50 μg/ml Ascorbic acid, 10 nM dexamethasone (Sigma, USA) until 21 days at 37 °C and 5% CO_2_; the medium was changed twice weekly. The experimental groups included three groups that received EVs including Cho-ag-EV, MSC-ag-EV, and Co-ag-EV in chondrogenic medium. All these groups were examined in the presence and absence of TGF ß1. The control group received a chondrogenic differentiation medium with TGF ß1. The micromasses size was calculated by ImageJ.

### RT-PCR analysis

To analyze the expressions of genes related to chondrogenesis (Acan, Col II, and Sox9) and Col X, we employed qRT-PCR. RNA extraction and synchronous synthesis of cDNA from the cell micro-mass were executed with a cell-to-cDNA kit. The interactions of qRT-PCR were performed with a Maxima SYBR Green/ROX qPCR Master Mix (Yekta Tajhiz, Iran) in an Applied Biosystems Step One Plus Real-time PCR system. The samples were prepared from 3 independent biological replicates. Each sample was replicated twice, and in addition, 2 negative controls were regarded for each primer. Supplementary Table 1 displays the primers that were employed for this evaluation.

### In vivo evaluation of isolated EVs on Osteoarthritis (OA) model

Adult male Wistar rats (weight ranging from 230 to 260 g) bought from the animal center of Royan institute (Tehran, Iran) were employed for all experiments. These rats were anaesthetized with ketamine and xylazine (90 mg/kg, 10 mg/kg) and allocated into different experimental and control groups randomly. Amongst them, 5 rats were in the intact group, and 20 rats were utilized to induce the OA model, by a single injection of 2 mg MIA/ 50 μl PBS per left knee^34^. After 3 weeks, the OA was confirmed by footprint and radiology, then the animal was utilized for experiments. Control groups included intact and without treatment or OA control (OA Ctrl) groups. Experimental groups included groups that received 50 μg EV/50 μl PBS (per left knee) from chondrocyte aggregate, MSC aggregate and MSCs/chondrocytes co-aggregates. Experimental groups received injections once a week for 4 weeks and were followed up for 8 weeks (Fig. 6B). All animals were maintained and used according to the National Institute of Health Guide for the Care and Use of Laboratory Animals and were approved by the Ethics Committee for Animal Experimentation of Royan Institute (code: IR.ACECR.ROYAN.REC.1397.113). This study is also reported in accordance with ARRIVE guidelines.

### Gait analysis (Footprints)

Footprints were collected using the paw print test, at 12 weeks after initiation of treatment of the OA model rats according to the protocol described previously^34^. Briefly, the ventral surface of the hind paws of the rats were stained with red ink. These rats were allowed to walk on a 100 cm-long, 8 cm-wide path covered with white paper. To attract the rats a dark chamber was placed at the end of the path. After the test, 3 sequential steps were applied to define the mean values for each measurement, including, stride length and step length for calculating irregularity. To determine the stride length (cm) we measured the distance of the same hind paw between two steps, and also measured the horizontal distance between the left and right paw as step length (cm), and the paw angle as the angle between the second toe and the calcaneus and a horizontal line (°).

### Radiography

To assess the severity of OA, the rats were put under general anesthesia (by a combination of xylazine 10 mg/kg body weight and ketamine 90 mg/kg), and radiography was performed using by a micro-X-ray. Radiography was performed in two positions including the dorsolateral and anteroposterior and the resulting image was scored according to a numerical rating scale.

### Histological analysis

Chondrogenic differentiation during in vitro study was evaluated by both toluidine blue and Safranin-o staining of micro-mass sections. All micromasses were fixed in 4% paraformaldehyde at 4 °C and immersed in paraffin and then sliced into 5 μm thick sections. The sections were stained after hydration, using toluidine blue or safranin-o for 30 s at room temperature for determining proteoglycan (PRGs) subunits in the extracellular matrix as an important characteristic of hyaline cartilage that could be investigated under the light microscope. Photography of slides was performed by an optical microscope (Olympus BX51; Olympus, Tokyo, Japan). For the histological study of the rats' knees, hind limbs were divaricated at the knee joint from sacrificed animals. After being fixed in 10% natural buffered formalin (NBF) solution for 48 h, all tissues were decalcified for 28 days by 10% (w/v) EDTA (pH 7.4). The decalcified samples were immersed in paraffin and cut into 5 μm sections. Subsequently, sections were stained with hematoxylin–eosin (H&E), toluidine blue, and safranin O. The stained histological sections were studied by a light microscope (Olympus BX51; Olympus, Tokyo, Japan) and scored by an independent pathologist by using Mankin’s scoring system blindly^60^.

### Immunohistochemical analysis

Analyses of immunohistochemistry (IHC) were performed to visualize the COL II (AB-2865 442, Thermofisher USA) contents of articular cartilage in the different groups. The joint sections were provided, and after dehydration with alcohol, antigen retrieval was carried out with 0.05% trypsin for 30 min at 37 °C. The blocking was performed with 1.5% goat serum in PBS and then treated with primary antibodies of rabbit anti-collagen type II (250,484, Abbiotec, USA) overnight at 4 °C. Then, they were incubated with anti-mouse (A2554, Sigma-Aldrich, Germany) and anti-rabbit (ab97051, Abcam, UK) secondary antibodies for one hour. Photographs were captured by an optical microscope (Olympus BX51; Olympus, Tokyo, Japan). The IHC images were captured at 200 × magnification from sections, and random areas of interest (ROIs) were obtained independently in the osteochondral region by a board-certified pathologist blinded to the grouping. Since the area of viable parts in these groups is restricted, five ROIs were imaged as available. The captured images were analyzed at 200 × magnification to quantify DAB expressions after import into the Image ProPlus software (Media Cybernetics, USA). The DAB positive area compared with the non-positive area for each ROI was expressed as a percentage value (DAB positive area %).

The same protocol was used to visualize COL I and II density in all micromasses. After 21 days of culture, the harvested micromasses were fixed in 4% paraformaldehyde at 4 °C, then they were cut with a thickness of 5 μm. Following dehydration with alcohol, antigen retrieval with 0.05% trypsin and blocking with 1.5% goat serum in PBS, samples were treated overnight at 4 °C with rabbit anti-collagen type I (sc-59772) or anti-collagen type II (250,484, Abbiotec, USA) primary antibodies, and they were incubated for one hour with anti-mouse (A2554, Sigma-Aldrich, Germany) secondary antibodies. Photographs were captured by an optical microscope (Olympus BX51; Olympus, Tokyo, Japan) and analyzed by Image ProPlus software (Media Cybernetics, USA) as mentioned above.

### Statistical analysis

Data analysis was carried out by t-test for two groups, and a one-way analysis of variance (one-way ANOVA) was used for comparison of the results between each group with Graph Pad Prism software (version 8.0.1). Data are displayed as mean ± SEM. The results were evaluated statistically significant at *p* < 0.05 for the comparison of in vitro and in vivo results.

### Ethics approval

All procedures were approved by the Animal Care and Ethics Committee at Royan Institute, Tehran, Iran (IR.ACECR.ROYAN.REC.1397.113).

## Supplementary Information

Supplementary Information.

## Data Availability

All data generated or analyzed during this study are included in this article. Further inquiries can be directed to the corresponding author.
