# Supplementary material for: Co-aggregation of MSC/chondrocyte in a dynamic 3D culture elevates the therapeutic effect of secreted extracellular vesicles on osteoarthritis in a rat model
Source: Sci Rep. 2022 Nov 18;12:19827. doi: 10.1038/s41598-022-22592-4 (PMC9674636; doi:10.1038/s41598-022-22592-4)
Supplement: Supplementary file 1 — Supplementary Information. [file 41598_2022_22592_MOESM1_ESM.docx]

***Supplementary Materials***

**Co-aggregation of MSC/Chondrocyte in a Dynamic 3D Culture Elevates the Therapeutic Effect of Secreted Extracellular Vesicles on Osteoarthritis in a Rat Model**

Abazar Esmaeili^1,2^, Samaneh Hosseini^1,3^, Amir Kamali^1^, Maryam Hosseinzadeh^1^, Faezeh Shekari^1^, Mohamadreza Baghaban Eslaminejad^1^*

^1^Department of Stem Cells and Developmental Biology, Cell Science Research Center, Royan Institute for Stem Cell Biology and Technology, ACECR, Tehran, Iran

^2^Faculty of Sciences and Advanced Technologies in Biology, University of Science and Culture, Tehran, Iran

^3^Department of Cell Engineering, Cell Science Research Center, Royan Institute for Stem Cell Biology and Technology, ACECR, Tehran, Iran

*Corresponding authors: Mohamadreza Baghaban Eslaminejad, e-mail: eslami@royaninstitute.org, Tel: +982123562524; Fax: +982123562507

Matured New Zealand white rabbits (Oryctolagus cuniculus) were purchased from the animal house of Royan Institute, Tehran, Iran. The Rabbits used in the experiments weighed nearly 3 kg (ranging from 2.5 to 3.5 Kg). The animals were preserved in agreement with the animal house guidelines and approval from the Ethics Committee of the Royan Institute. We used one dose of an intramuscular injection of 35 mg/kg ketamine and 10 mg/kg xylazine mixture (ketamine HCL 100 mg/ml and xylazine HCL 20 mg/ml, Alfasan, Holland) for anesthetizing of rabbits. Then we used them for bone marrow aspiration. The animals were retained in one cage while they were free to move.

***MSCs isolation and culture***

Bone marrow from Tibia bone in rabbits was aspirated and centrifuged. Isolated cells were cultured in DMEM medium containing 15% FBS serum, 1% Pen/Strep and stored in an incubator at 37 ° C with humidified air and 5% CO_2_. After observing the MSC colonies, they were transferred to a new culture flask after reaching about 80% confluency. The cells in each passage were more purified, and the cells in the third passage were used for further experiments.

***MSC differentiation assay***

In order to verify the mesenchymal phenotype of the isolated cells, passage-3 cells were differentiated into adipogenic, and osteogenic lineages. 0.3×10^6^ cells were seeded per well of a 6-well culture plate. For osteogenic differentiation, the medium was changed by osteogenic medium-DMEM supplemented with 50 mg/ml ascorbic acid 2- phosphate (Sigma, USA), 10 mM β-glycerol phosphate (Sigma, USA) and 10 nM dexamethasone (Sigma, USA). After 3 weeks, the medium was removed and the cell monolayers were fixed in methanol, and after, staining with alizarin red, investigated under the light microscope.

For adipogenic differentiation, the adipogenic medium that contained 100 nM dexamethasone (Sigma, USA) and 50 mg/ml indomethacin (Sigma, USA), 100 μM L-Ascorbic acid (Sigma, USA) was added to each well. On day 21, the culture medium was discarded and the cells were fixed in 4% formalin at room temperature for 1 hour, and after staining with oil red solution in isopropanol 99% for 15 minutes, they were investigated under the light microscope for the adipose droplets observation.

Chondrogenic differentiation of MSCs was also performed. We operated a micro-mass culture system to enforce chondrogenic differentiation of the isolated MSCs. Brieflly, 2.5 ×10^6^ passage-3 rabbit bone marrow MSCs were cultured in a 96 well plate for micro-mass formation in a chondrogenic medium (as described in the *in vitro* study). The cells were preserved in this medium for 21 days at 37°C and 5% CO2, with twice-weekly medium changes. Chondrogenic differentiation was evaluated by Safranin O and Toluidine Blue staining of the pellet sections.

***Chondrocyte isolation and culture***

Knee joints of sacrificed rabbits were removed in agreement with the ethical principles of working with laboratory animals. The sample was transferred to ice in the laboratory under aseptic conditions, and under sterile conditions with the help of a surgical razor, the cartilage layer was completely separated from the knee condyle. After crushing, 2-3 mm pieces of cartilage tissue were treated with 0.8% Collagenase type I (Sigma-Aldrich, USA) and incubated overnight at 37 ° C. The chondrocytes isolated from the tissue were centrifuged at 1200 rpm for 5 minutes and cultured in Dulbecco’s Modified Eagle Medium (DMEM, +4500 mg/L Glucose, Gibco, USA) containing 10% fetal bovine serum (FBS, Gibco, USA), 1% Pen/Strep (50 U/ml penicillin+50 μg/ml streptomycin, Gibco, USA) and stored in a humidified incubator at 37 °C with 5% CO2. The cell culture medium was changed every 3 days and cells were transferred to new flasks once a week.


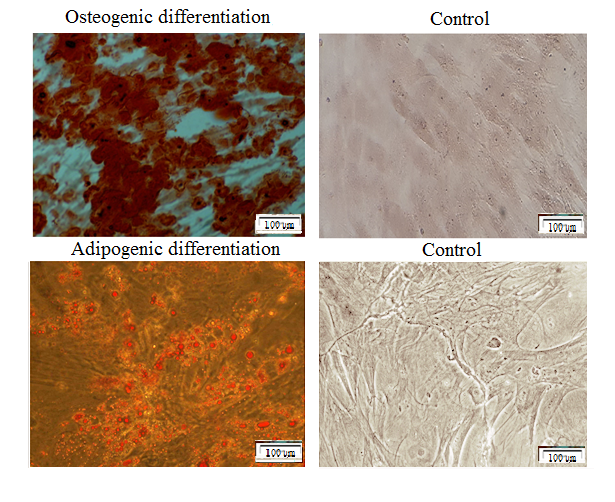


Figure 1S– Osteogenic (above) and adipogenic (below) differentiation of MSCs derived from rabbit bone marrow (Scale bars= 100 μm).


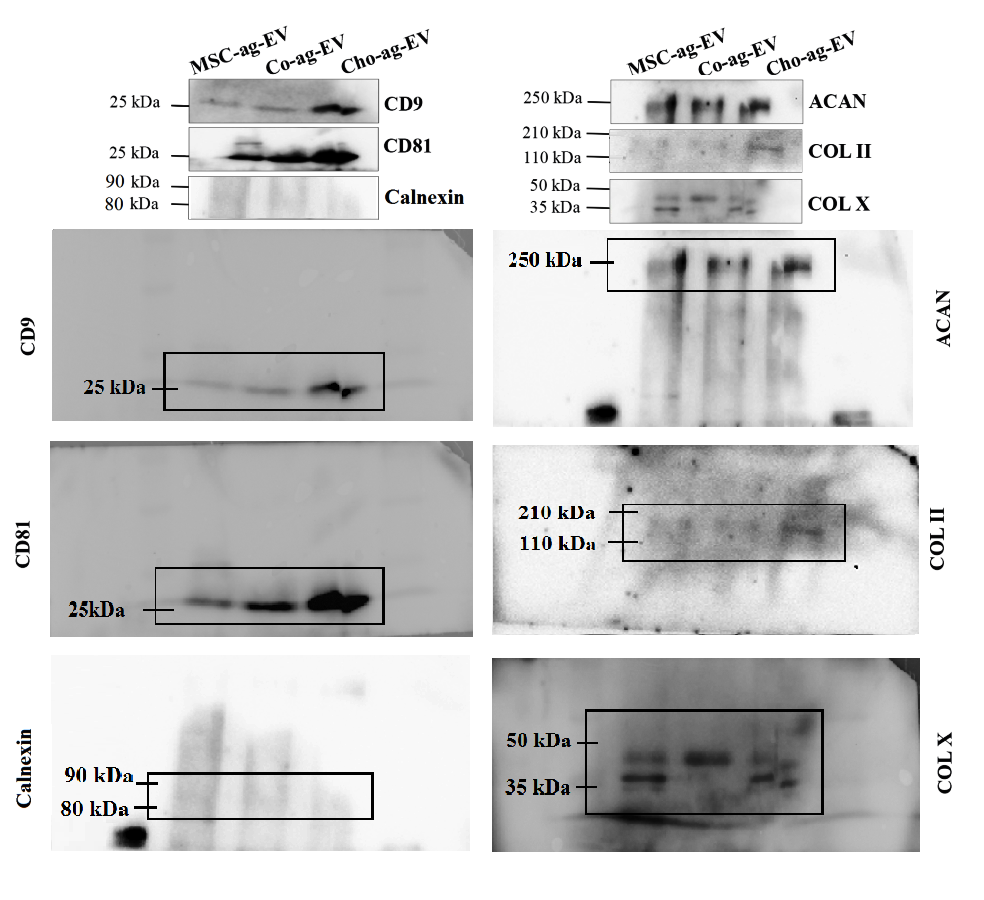


**Figure 2S**: Full-length blots related to Figure 2C were included in this figure. All these images are original and unprocessed. The blots' bands have been clarified with a substrate (Super Signal West Femto PI3 4096, Thermo Fisher, USA) (ECL Prime Western Blotting Detection Reagents, RPN2232 Sigma-Aldrich, USA). We utilized the pre-Stained Protein Marker after the gel-dock device because of the high sensitivity. All blots were imaged with the Aliens Q9 Advanced Chemoluminescence Imager, UVITEC.

Table 1S: List of primers used in qPCR for chondrogenic genes.

| Genes | Primer sequences |
| --- | --- |
| Col X | F: GAACCCAGAATCCATCTGAG  R: GGCATAGGGAATGAAGAACTG |
| Sox9 | F: AGTAGGCAATAGTGTAGAGGAC  R: CGGTGTTTAAGGCTCAAGG |
| Col II | F: CAAGTCCCTCAACAACCAG  R: TATCCAGTAGTCACCGCTC |
| Acan | F: TGCCACTGTGAGAGTTCC  R: ACATTCCACACCCAGAGTT |
